# On the spectral radius and energy of signless Laplacian matrix of digraphs

**DOI:** 10.1016/j.heliyon.2022.e09186

**Published:** 2022-03-28

**Authors:** Hilal A. Ganie, Yilun Shang

**Affiliations:** a Department of School Education, JK Govt. Kashmir, India; b Department of Computer and Information Sciences, Northumbria University, Newcastle NE1 8ST, UK

**Keywords:** Digraphs, Strongly connected digraphs, Signless Laplacian spectral radius, Generalized adjacency spectral radius, Energy

## Abstract

Let *D* be a digraph of order *n* and with *a* arcs. The signless Laplacian matrix Q(D) of *D* is defined as Q(D)=Deg(D)+A(D), where A(D) is the adjacency matrix and Deg(D) is the diagonal matrix of vertex out-degrees of *D*. Among the eigenvalues of Q(D) the eigenvalue with largest modulus is the signless Laplacian spectral radius or the *Q*-spectral radius of *D*. The main contribution of this paper is a series of new lower bounds for the *Q*-spectral radius in terms of the number of vertices *n*, the number of arcs, the vertex out-degrees, the number of closed walks of length 2 of the digraph *D*. We characterize the extremal digraphs attaining these bounds. Further, as applications we obtain some bounds for the signless Laplacian energy of a digraph *D* and characterize the extremal digraphs for these bounds.

## 1 Introduction

Let D=(V(D),E(D)) be a digraph, where $V(D)=\{v_{1},v_{2},\ldots ,v_{n}\}$ is the set of vertices and E(D) is the set of arcs in *D*. A digraph *D* is called a simple digraph if there are no loops or multiple arcs. A digraph *D* is called strongly connected if any two vertices $v_{i},v_{j}\in V(D)$ can be connected by directed paths from $v_{i}$ to $v_{j}$ and vice versa. A digraph is considered as connected if its undirected version is connected as a graph. Throughout this paper, we confine ourselves to connected simple digraphs. For any notions not defined explicitly in the paper we will refer the reader to the standard book [9].

For two vertices *u* and *v* in a digraph *D*, if there is an arc (u,v)∈E(D) or (v,u)∈E(D), they are called adjacent. If both arcs (u,v),(v,u)∈E(D), the two vertices are called doubly adjacent. Given an arc $e=(v_{i},v_{j})\in E(D)$, we call $v_{i}$ the initial vertex of *e*, $v_{j}$ the terminal vertex, and $v_{i}$ a tail of $v_{j}$. The in-neighborhood and out-neighborhood of $v_{i}$ is denoted, respectively, by $N_{D}^{-}(v_{i})=\{v_{j}\in V(D)|(v_{j},v_{i})\in E(D)\}$ and $N_{D}^{+}(v_{i})=\{v_{j}\in V(D)|(v_{i},v_{j})\in E(D)\}$. Accordingly, the in-degree and out-degree are denoted by $d_{i}^{-}=|N_{D}^{-}(v_{i})|$ and $d_{i}^{+}=|N_{D}^{+}(v_{i})|$, respectively. Let $\delta^{+}$ be the minimum out-degree and $\Delta^{+}$ be the maximum out-degree. Similarly, let $\delta^{-}$ be the minimum in-degree and $\Delta^{-}$ be the maximum in-degree. *D* is called out-degree regular if $d_{1}^{+}=d_{2}^{+}=\cdots =d_{n}^{+}$.

Let $\pi :u=u_{0},u_{1},\ldots ,u_{l}=v$ be a sequence of vertices, where $\left(u_{k-1},u_{k}\right)$ forms an arc in *D* for any 1≤k≤l. *π* is called a walk of length *l* from *u* to *v*. *π* is a closed walk if u=v. Write $c_{2}^{\left(i\right)}$ for the number of closed walks of length 2 associated with the vertex $v_{i}\in V(D)$. The sequence $\left(c_{2}^{\left(1\right)},c_{2}^{\left(2\right)},\ldots ,c_{2}^{\left(n\right)}\right)$ is a closed walk sequence of length 2 in *D*. Clearly, we know that $c_{2}=c_{2}^{\left(1\right)}+c_{2}^{\left(2\right)}+\ldots +c_{2}^{\left(n\right)}$ is equivalent to the number of closed walks of length 2.

A digraph *D* is symmetric if the existence of any arc (u,v)∈E(D) implies the existence of the other one (v,u)∈E(D). It is easy to see that any simple graph naturally corresponds to a symmetric digraph by following the mapping $G\to \overset{↔}{G}$, where $\overset{↔}{G}$ and *G* share the same vertex set and each edge *uv* in *G* is mapped to the arcs (u,v) and (v,u).

Write *D* for a digraph having the adjacency matrix $A(D)=(a_{ij})$, where $a_{ij}=1$ if $\left(v_{i},v_{j})\in E(D\right)$ and $a_{ij}=0$ otherwise. The diagonal matrix of out-degrees is denoted by $Deg(D)=(d_{1}^{+},d_{2}^{+},\ldots ,d_{n}^{+})$. The signless Laplacian matrix Q(D) of the digraph *D* is defined as Q(D)=Deg(D)+A(D). Clearly, Q(D) is a real non-negative matrix, which is not necessarily symmetric. The signless Laplacian eigenvalues of the digraph *D*, denoted by $q_{1}(D),q_{2}(D),\ldots ,q_{n}(D)$, are the eigenvalues of Q(D). The signless Laplacian spectral radius or *Q*-spectral radius, denoted by $q_{1}(D)=q(D)$, is the eigenvalue that has the largest modulus [6]. When *D* forms a strongly connected digraph, an immediate application of the Perron-Frobenius Theorem [16] implies that q(D) is an eigenvalue of Q(D) and q(D) admits a unique positive unit eigenvector. This eigenvector is the so-called Perron vector of Q(D). The signless Laplacian spectral radius of digraphs have attracted considerable attention in the algebraic graph theory and as such various papers have been published featuring the bounds and extremal results. Some recent results in this direction have been reported in for example [1], [2], [7], [10], [13], [15], [19], [22] and the references therein.

For a digraph *D* with *n* vertices and *a* arcs, the signless Laplacian energy is denoted by $E_{SL}(D)$ and is defined in [23] as

$$E_{SL}(D)=\sum_{i=1}^{n}|q_{i}(D)-\frac{a}{n}|=\sum_{i=1}^{n}|\alpha_{i}|,$$

where $\alpha_{i}=q_{i}(D)-\frac{a}{n}$ and $q_{1},q_{2},\ldots ,q_{n}$ are the signless Laplacian eigenvalues of *D*. For some bounds on the signless Laplacian energy of a digraph, we refer to [5], [23].

The rest of the paper is organised as follows. In Section 2, we obtain some new lower bounds for the *Q*-spectral radius in terms of the number of vertices *n*, the number of arcs, the vertex out-degrees, the number of closed walks of length 2 of the digraph *D*. We characterize the extremal digraphs attaining these bounds. Further, as applications we obtain some bounds for the signless Laplacian energy of a digraph *D* and characterize the extremal digraphs attaining these bounds.

## 2 Signless Laplacian spectral radius

Given a nonnegative matrix $A=(a_{ij})\in R^{n\times n}$, its geometric symmetrization is given by $S(A)=(s_{ij})\in R^{n\times n}$, where $s_{ij}=\sqrt{a_{ij}a_{ji}}$ for i,j=1,2,…,n. Let λ(M) be the spectral radius of the matrix *M*. The spectral radius of the matrices *A* and S(A) satisfies [18] λ(A)≥λ(S(A)).

For a digraph *D* of order *n* with *a* arcs, we denote by Q(D) its signless Laplacian matrix. The geometric symmetrization of Q(D) is given by $S(Q(D))=(s_{ij})$. Clearly, we have $\sum_{j=1}^{n}s_{ij}=d_{i}^{+}+c_{2}^{\left(i\right)}$ for any vertex $v_{i}\in V(D)$.

The following lemma is a result in [16].

Lemma 2.1 *Let A and B be nonnegative matrices with their respective spectral radii*λ(A)*and*λ(B)*. If*0≤A≤B*, then*λ(A)≤λ(B)*. Furthermore, if B is irreducible and*0≤A<B*, then*λ(A)<λ(B)*.*

The following result gives a lower bound for the signless Laplacian spectral radius of a digraph.

Theorem 2.2 *Let D digraph of order n having a arcs. Let*$Q^{2}=(q_{ij}^{⁎})$*be the square of the signless Laplacian matrix and let*$S(Q^{2})=(s_{ij}^{⁎})$*be the geometric symmetrization of*$Q{\left(D\right)}^{2}$*. Then*

(2.1) $$q(D)\geq \sqrt{\frac{\sum_{i=1}^{n}\sum_{j=1}^{n}s_{ij}^{⁎}}{n}}.$$

*For a strongly connected digraph D, equality occurs in*(2.1)*if and only if*$D=\overset{↔}{G}$*with each connected component of D a r-regular graph such that*$r^{2}=\frac{\sum_{i=1}^{n}\sum_{j=1}^{n}s_{ij}^{⁎}}{n}$*.*

Proof Let $S(Q^{2})=(s_{ij}^{⁎})$ be the geometric symmetrization of $Q^{2}$. Therefore $Q^{2}\geq S(Q^{2})\geq 0$. In the light of Lemma 2.1, we obtain $\lambda (Q^{2})\geq \lambda (S(Q^{2}))$. Noting that the matrix $S(Q{\left(D\right)}^{2})$ is symmetric, via Rayleigh quotient, we obtain for $X=e={\left(1,1,\ldots ,1\right)}^{T}$, the all one *n*-column vector, and that

(2.2) $$\lambda (Q(D))=\sqrt{\lambda (Q^{2})}\geq \sqrt{\lambda (S(Q^{2}))}=\sqrt{\max_{X\neq 0}\frac{X^{T}S(Q^{2})X}{X^{T}X}}\geq \sqrt{\frac{e^{T}S(Q^{2})e}{e^{T}e}}=\sqrt{\frac{\sum_{i=1}^{n}\sum_{j=1}^{n}s_{ij}^{⁎}}{n}}.$$

This proves the inequality (2.1). If the equality in (2.1) is true, the above involved inequalities will become equalities. Using the equality in (2.2), it is clear that $\lambda (Q^{2})=\lambda (S(Q^{2}))$ and $\lambda (S(Q^{2}))=\frac{e^{T}S(Q^{2})e}{e^{T}e}$. The second equality indicates that *e* is an eigenvector of $S(Q^{2})$ associated with the eigenvalue $\lambda (S(Q^{2}))$. Hence, the multiplicity of the eigenvalue $\lambda (S(Q^{2}))$ can be one or two. If *D* is strongly connected, Q(D) becomes irreducible and $Q^{2}$ is irreducible too. Recall that $Q^{2}\geq S(Q^{2})$ and Q(D) is an irreducible matrix. If $Q^{2}>S(Q^{2})$, invoking Lemma 2.1 we know that $\lambda (Q^{2})>\lambda (S(Q^{2}))$. This is a contradiction to the assumption of equality. Consequently, we proved that $Q^{2}=S(Q^{2})$, which means Q(D) is symmetric and hence $D=\overset{↔}{G}$. If the multiplicity of $\lambda (S(Q^{2}))$ is one, $Q^{2}=S(Q^{2})$ is symmetric and $\lambda (Q^{2})=\lambda^{2}(Q(D))$. Noting that *e* is an eigenvector associated with the eigenvalue $\lambda (Q^{2})$, we know that λ(Q(D)) is an eigenvalue of Q(D) associated with eigenvector *e*. This suggests that *D* is a *r*-regular graph satisfying $r^{2}=\frac{\sum_{i=1}^{n}\sum_{j=1}^{n}s_{ij}^{⁎}}{n}$. Therefore, the equality holds true when $D=\overset{↔}{G}$ and *D* is *r*-regular satisfying $r^{2}=\frac{\sum_{i=1}^{n}\sum_{j=1}^{n}s_{ij}^{⁎}}{n}$. On the other hand, if the multiplicity of $\lambda (S(Q^{2}))$ is two, both λ(S(Q(D))) and −λ(S(Q(D))) are eigenvalues of Q(D). This suggests that some of the eigenvalues of Q(D) must be negative. Note that $D=\overset{↔}{G}$ implies that Q(D) coincides with the signless Laplacian matrix Q(G) of the graph in question. In view of the fact that Q(D) is positive semi-definite, this case is false.

Suppose that *D* is the direct sum of its disjoint strongly connected components $D_{1},D_{2},\ldots ,D_{s}$. Denote by $Q(D_{k})\in R^{n_{k}\times n_{k}}$ the signless Laplacian matrix of the component $D_{k}$ satisfying $\sum_{k=1}^{n}n_{k}=n$. We obtain

$$Q^{2}(D)=\left(\begin{array}{cccc} Q^{2}(D_{1})\; & \; & \; & \\ \; & Q^{2}(D_{2})\; & \; & \\ \; & \; & \ddots \;\\ \; & \; & \; & Q^{2}(D_{s}) \end{array}\right),$$

where the unspecified elements are zeros. Note that $S(Q^{2})$ is a block diagonal matrix. As $S(Q^{2})$ is symmetric, we obtain $\lambda (S(Q^{2}(D)))=\max_{k}\lambda (S(Q^{2}(D_{k})))$. Let $e_{k}$ be the all one column vector of order $n_{k}$. Since the equality in (2.1) holds true, we obtain

$$\lambda (Q(D))=\sqrt{\lambda (Q^{2})}\geq \sqrt{\lambda (S(Q^{2}))}=\sqrt{\max_{X\neq 0}\frac{X^{T}S(Q^{2})X}{X^{T}X}}\;\;=\sqrt{\frac{e^{T}S(Q^{2})e}{e^{T}e}}=\sqrt{\sum_{k=1}^{s}\frac{n_{k}\lambda (S(Q^{2}(D_{k})))}{n}}\leq \sqrt{\max_{k}\lambda (S(Q^{2}(D_{k})))}\;\;=\max_{k}\sqrt{\lambda (S(Q^{2}(D_{k})))}=\sqrt{\lambda (S(Q^{2}(D)))}=\sqrt{\lambda (Q^{2}(D))}=\lambda (Q(D)),$$

which means for every k=1,2,…,s,

$$\lambda (Q(D))=\sqrt{\lambda (Q^{2}(D))}=\sqrt{\lambda (Q^{2}(D_{k}))}=\sqrt{\lambda (S(Q^{2}(D_{k})))}=\sqrt{\sum_{k=1}^{s}\frac{e_{n_{k}}^{T}S(Q^{2}(D_{k}))e_{n_{k}}}{n_{k}}}.$$

As a result, $D_{k}=\overset{↔}{G_{k}}$ is a symmetric digraph, in which every connected component $G_{k}$ is a *r*-regular graph. This completes the proof. □

For any α∈[0,1], the generalized adjacency matrix $A_{\alpha }(D)$ of a digraph *D* is given by

$$A_{\alpha }(D)=\alpha Deg(D)+(1-\alpha )A(D).$$

We have $A_{\alpha }(D)=A(D)$ if α=0, $2A_{\alpha }(D)=Q(D)$ if $\alpha =\frac{1}{2}$, and $A_{\alpha }(D)=Deg(D)$ if α=1. It turns out that the matrix $A_{\alpha }(D)$ unifies the spectral theory of the adjacency matrix A(D) and the signless Laplacian matrix Q(D) of a digraph *D*. Let $\lambda_{1}(A_{\alpha }(D)),\lambda_{2}(A_{\alpha }(D)),\ldots ,\lambda_{n}(A_{\alpha }(D))$ be the eigenvalues of $A_{\alpha }(D)$. They are often referred to as the generalized adjacency eigenvalues or the $A_{\alpha }$-eigenvalues of *D*. The matrix $A_{\alpha }(D)$ is not symmetric in general and may have complex spectra. Let $\lambda_{1}(A_{\alpha }(D))=\lambda (A_{\alpha }(D))$ be the eigenvalue of $A_{\alpha }(D)$ with largest modulus. It is often called the generalized adjacency spectral radius or $A_{\alpha }$-spectral radius of digraph *D*. For some recent papers regarding the spectral properties of generalized adjacency matrix, we refer to [3], [4], [11], [12], [20], [21], [24] and the references therein.

Using the concept of geometric symmetrization and proceeding similar to Theorem 2.2, the following lower bounds (Theorem 2.3 and Theorem 2.5) were obtained in [11].

Theorem 2.3 *Let D be a digraph of order n with a arcs. Suppose that*α∈[0,1)*. Denote by*$\left(c_{2}^{\left(1\right)},c_{2}^{\left(2\right)},\ldots ,c_{2}^{\left(n\right)}\right)$*the sequence of closed walks of length* 2*. We have*

(2.3) $$\lambda (A_{\alpha }(D))\geq \frac{\alpha a+(1-\alpha )c_{2}}{n},$$

*where* $c_{2}=c_{2}^{\left(1\right)}+c_{2}^{\left(2\right)}+\cdots +c_{2}^{\left(n\right)}$ *is the number of closed walks of length* 2 *in D. When* α=0*, the equality in* (2.3) *holds true if and only if* $D=\overset{↔}{G}+$*{possibly some arcs that do not belong to cycles}, where every connected component of G is r-regular satisfying* $r=\frac{c_{2}}{n}$*. If* α≠0*, for a strongly connected digraph D, the equality in* (2.3) *holds true if and only if* $D=\overset{↔}{G}$*, in which every connected component of G is r-regular satisfying* $r=\frac{\alpha a+(1-\alpha )c_{2}}{n}$*.*

Taking $\alpha =\frac{1}{2}$ in Theorem 2.3 and using the fact that $Q(D)=2A_{\frac{1}{2}}(D)$, we get the following lower bound for the signless Laplacian spectral radius q(D) in terms of the number of arcs, the number of closed walks and the order of the digraph *D*.

Theorem 2.4 *Let D be a digraph of order n with a arcs. Suppose that*$\left(c_{2}^{\left(1\right)},c_{2}^{\left(2\right)},\ldots ,c_{2}^{\left(n\right)}\right)$*is the sequence of closed walks of length* 2*. We obtain*

(2.4) $$q(D)\geq \frac{a+c_{2}}{n},$$

*where* $c_{2}=c_{2}^{\left(1\right)}+c_{2}^{\left(2\right)}+\cdots +c_{2}^{\left(n\right)}$ *is the number of closed walks of length* 2 *in D. For a strongly connected digraph D, the equality in* (2.4) *holds true if and only if* $D=\overset{↔}{G}$*, in which every connected component of G is r-regular satisfying* $r=\frac{a+c_{2}}{n}$*.*

Another lower bound for the generalized adjacency spectral radius is obtained in [11].

Theorem 2.5 *Let D be a digraph of order n with a arcs. Suppose that*α∈[0,1)*. Denote by*$\left(c_{2}^{\left(1\right)},c_{2}^{\left(2\right)},\ldots ,c_{2}^{\left(n\right)}\right)$*the sequence of closed walks of length* 2*. We obtain*

(2.5) $$\lambda (A_{\alpha }(D))\geq \sqrt{\frac{\sum_{i=1}^{n}{\left(\alpha d_{i}^{+}+(1-\alpha )c_{2}^{\left(i\right)}\right)}^{2}}{n}}.$$

*When* α=0*, the equality in* (2.4) *holds true if and only if* $D=\overset{↔}{G}+$*{possibly some arcs that do not belong to cycles}, in which every connected component of G is r-regular or* $(r_{1},r_{2})-$ *semiregular bipartite, satisfying* $r^{2}=r_{1}r_{2}=\frac{\sum_{i=1}^{n}{\left(c_{2}^{\left(i\right)}\right)}^{2}}{n}$*. When* α≠0*, for a strongly connected digraph D, the equality in* (2.5) *holds true if and only if* $D=\overset{↔}{G}$ *with every connected component of G being r-regular with* $r^{2}=\frac{\sum_{i=1}^{n}{\left(\alpha d_{i}^{+}+(1-\alpha )c_{2}^{\left(i\right)}\right)}^{2}}{n}$ *or* $D=\overset{↔}{G}$ *with every connected component of G having the property that* $\lambda (A_{\alpha }(D))$ *and* $-\lambda (A_{\alpha }(D))$ *are the eigenvalues of* $A_{\alpha }(D)$ *associated with the eigenvector* $e={\left(1,1,\ldots ,1\right)}^{T}$*.*

Taking $\alpha =\frac{1}{2}$ in Theorem 2.5 and using the fact that $Q(D)=2A_{\frac{1}{2}}(D)$, we get the following lower bound for the signless Laplacian spectral radius q(D) in terms of the number of arcs, the number of closed walks and the order of the digraph *D*.

Theorem 2.6 *Let D be a digraph of order n with a arcs. Suppose that*$\left(c_{2}^{\left(1\right)},c_{2}^{\left(2\right)},\ldots ,c_{2}^{\left(n\right)}\right)$*is the sequence of closed walks of length* 2*. We have*

(2.6) $$q(D)\geq \sqrt{\frac{\sum_{i=1}^{n}{\left(d_{i}^{+}+c_{2}^{\left(i\right)}\right)}^{2}}{n}},$$

*where* $c_{2}=c_{2}^{\left(1\right)}+c_{2}^{\left(2\right)}+\cdots +c_{2}^{\left(n\right)}$ *is the number of closed walks of length* 2 *in D. For a strongly connected digraph D, the equality in* (2.6) *holds true if and only if* $D=\overset{↔}{G}$ *with every connected component of G being r-regular with* $r^{2}=\frac{\sum_{i=1}^{n}{\left(d_{i}^{+}+c_{2}^{\left(i\right)}\right)}^{2}}{n}$*.*

The following Lemma was obtained in [8].

Lemma 2.7 *Let*$A\in R^{n\times n}$*be a nonnegative matrix. Let*S(A)*be the geometric symmetrization of A. We have*$S(A^{2})\geq S{\left(A\right)}^{2}$*, where the equality holds true if and only if A is symmetric.*

Remark 2.8 Using Lemma 2.7, it is clear that the lower bound given by Theorem 2.2 is sharper than that shown in Theorem 2.4.

## 3 Bounds for the signless Laplacian energy

In this section, we obtain some new bounds for the signless Laplacian energy of a digraph *D* in terms of different parameters associated with the structure of the digraph. We characterize the extremal digraphs attaining these bounds.

The first Zagreb index of a graph *G* is denoted by Zg(G) and is defined as $Zg(G)=\sum_{i=1}^{n}d_{i}^{2}$, where $d_{i}$ is the degree of the *i*-th vertex of *G*. Likewise, we define the first out-degree Zagreb index, denoted by $Zg^{+}(D)$ of a digraph *D* as $Zg^{+}(G)=\sum_{i=1}^{n}{\left(d_{i}^{+}\right)}^{2}$ and the first in-degree Zagreb index of a digraph *D* as $Zg^{-}(G)=\sum_{i=1}^{n}{\left(d_{i}^{-}\right)}^{2}$.

The following result gives an upper bound for the signless Laplacian energy of a digraph *D*, in terms of the order, the number of arcs, the maximum out-degree, the first out-degree Zagreb index and the number of closed walks of length 2.

Theorem 3.1 *Let D be a digraph of order n having a arcs. Let*$\Delta^{+}$*be the maximum out-degree,*$Zg^{+}(D)$*be the first out-degree Zagreb index and*$c_{2}$*be the number of closed walks of length* 2 *of D. Then*

(3.1) $$E_{SL}(D)\leq 2\Delta^{+}-\frac{a}{n}+\sqrt{\left(n-1)(Zg^{+}(D)+a(1-\frac{a}{n})-{\left(\frac{c_{2}}{n}\right)}^{2}\right)}.$$

*For a strongly connected digraph D, equality occurs in* (3.1) *if and only if* $D={\overset{↔}{K}}_{n}$ *or* $D=\overset{↔}{G}$ *is* $\Delta^{+}$*-regular digraph with three distinct signless Laplacian eigenvalues, given by* $q(D)=2\Delta^{+},\frac{a}{n}+\theta$ *and* $\frac{a}{n}-\theta$*, where* $\theta =\sqrt{\frac{Zg^{+}(D)+a(1-\frac{a}{n})-{\left(\frac{c_{2}}{n}\right)}^{2}}{n-1}}$*.*

Proof Let $Q(D)=(q_{ij})$ be the signless Laplacian matrix of *D*. By Schur's triangularization theorem [16], there exists a unitary matrix *U* such that $U^{⁎}Q(D)U=T$, where $T=(t_{ij})$ is an upper triangular matrix with diagonal entries $t_{ii}=q_{i},\;i=1,2,\ldots ,n$. Therefore,

$$\sum_{i,j=1}^{n}|q_{ij}{|}^{2}=\sum_{i,j=1}^{n}|t_{ij}{|}^{2}\geq \sum_{i=1}^{n}|t_{ii}{|}^{2}=\sum_{i=1}^{n}|q_{i}{|}^{2},$$

that is,

$$\sum_{i=1}^{n}|q_{i}{|}^{2}\leq \sum_{i,j=1}^{n}|q_{ij}{|}^{2}=\sum_{i=1}^{n}{\left(d_{i}^{+}\right)}^{2}+a=Zg^{+}(D)+a,$$

where $Zg^{+}(D)=\sum_{i=1}^{n}{\left(d_{i}^{+}\right)}^{2}$ is the first out-degree Zagreb index of *D*. Now, proceeding similarly as in [23] (see inequality (8) onwards), we get

(3.2) $$\sum_{i=1}^{n}|\alpha_{i}{|}^{2}\leq a+\sum_{i=1}^{n}{\left(d_{i}^{+}-\frac{a}{n}\right)}^{2}=Zg^{+}(D)+a(1-\frac{a}{n}).$$

Since Q(D) is a non-negative matrix, therefore q(D) is an eigenvalue of Q(D). Let $q(D)=q_{1},q_{2},\ldots ,q_{n}$ be the signless Laplacian eigenvalues of *D* and let $\alpha_{i}=q_{i}-\frac{a}{n}$, for i=1,2,…,n. Applying the Cauchy-Schwarz inequality to the vectors $\left(|\alpha_{2}|,|\alpha_{3}|,\ldots ,|\alpha_{n}|\right)$ and (1,1,…,1) of $R^{n-1}$, we obtain

$${\left(\sum_{i=2}^{n}|\alpha_{i}|\right)}^{2}\leq (n-1)\sum_{i=2}^{n}|\alpha_{i}{|}^{2},$$

that is,

$${\left(E_{SL}(D)-|\alpha_{1}|\right)}^{2}\leq (n-1)(\sum_{i=1}^{n}|\alpha_{i}{|}^{2}-|\alpha_{1}{|}^{2}).$$

Using the inequality (3.2), we get

(3.3) $$E_{SL}(D)\leq |\alpha_{1}|+\sqrt{\left(n-1)(Zg^{+}(D)+a(1-\frac{a}{n})-|\alpha_{1}{|}^{2}\right)}.$$

Since Q(D) is a non-negative matrix and for a non-negative matrix spectral radius lies between the minimum and the maximum row sums, it follows that

(3.4) $$2\delta^{+}\leq q(D)\leq 2\Delta^{+},$$

with equality for a strongly connected digraph if and only if *D* is a $\Delta^{+}$-out-degree regular digraph. So, we have $|\alpha_{1}|=\alpha_{1}=q(D)-\frac{a}{n}\leq 2\Delta^{+}-\frac{a}{n}$. By Theorem 2.4, we have $q(D)\geq \frac{a+c_{2}}{n}$, giving that $|\alpha_{1}|=q(D)-\frac{a}{n}\geq \frac{c_{2}}{n}$. With these observations, it follows from (3.3) that

$$E_{SL}(D)\leq 2\Delta^{+}-\frac{a}{n}+\sqrt{\left(n-1)(Zg^{+}(D)+a(1-\frac{a}{n})-{\left(\frac{c_{2}}{n}\right)}^{2}\right)}.$$

The equality in (3.1) holds true if and only if

(i) $T=(t_{ij})$ is a diagonal matrix,
(ii) $\left|\alpha_{2}|=|\alpha_{3}|=\cdots =|\alpha_{n}\right|$,
(iii) the equality in $q(D)\leq 2\Delta^{+}$ holds true and
(iv) the equality in $q(D)\geq \frac{a+c_{2}}{n}$ holds true.

Thanks to Schur's unitary triangularization theorem [16], we have that $T=(t_{ij})$ is diagonal if and only if Q(D) is normal. By (3.4), the equality for a strongly connected digraph *D* in $q(D)\leq 2\Delta^{+}$ holds true, if and only if *D* is a $\Delta^{+}$-out-degree regular digraph. By Theorem 2.4 the equality for a strongly connected digraph *D* in $q(D)\geq \frac{a+c_{2}}{n}$ holds true, if and only if $D=\overset{↔}{G}$, where each connected component of *D* is a *r*-regular graph with $r=\frac{a+c_{2}}{n}$. Combining these observations it follows that the equality in (3.1) holds true if and only if $D=\overset{↔}{G}$, where each connected component of *G* is a $\Delta^{+}$-regular digraph and $\left|\alpha_{2}|=|\alpha_{3}|=\cdots =|\alpha_{n}\right|$. If $D=\overset{↔}{G}$, then each of $q_{i}(D)$ is a real number and so using the fact $\alpha_{n}=q_{n}(D)-\frac{a}{n}<0$ and $\alpha_{2}=q_{2}(D)-\frac{a}{n}\geq 0$ or $\alpha_{2}=q_{2}(D)-\frac{a}{n}<0$. If $\alpha_{2}=q_{2}(D)-\frac{a}{n}<0$, then $\left|\alpha_{2}|=|\alpha_{3}|=\cdots =|\alpha_{n}\right|$ gives that $q_{2}(D)=\cdots =q_{n}(D)$ and so the equality holds if *D* is symmetric $\Delta^{+}$-regular digraph with two distinct eigenvalues. Using a well-known fact that a connected graph *G* has two distinct signless Laplacian eigenvalues if and only if $G≅K_{n}$, it follows that equality occurs in this case if and only if $D={\overset{↔}{K}}_{n}$. If $\alpha_{2}=q_{2}(D)-\frac{a}{n}\geq 0$, then $\left|\alpha_{2}|=|\alpha_{3}|=\cdots =|\alpha_{n}\right|$ gives that their exists a positive integer *t*, such that $q_{2}(D)-\frac{a}{n}=\cdots =q_{t}(D)-\frac{a}{n}=\theta$ and $q_{t+1}(D)-\frac{a}{n}=\cdots =q_{n}(D)-\frac{a}{n}=-\theta$. That is, $q_{2}(D)=\cdots =q_{t}(D)=\frac{a}{n}+\theta$ and $q_{t+1}(D)=\cdots =q_{n}(D)=\frac{a}{n}-\theta$. Using the fact that for the digraph $D=\overset{↔}{G}$, we have $\sum_{i=1}^{n}|\alpha {|}^{2}=Zg^{+}(D)+a(1-\frac{a}{n})$, it is easy to verify that $\theta =\sqrt{\frac{Zg^{+}(D)+a(1-\frac{a}{n})-{\left(\frac{c_{2}}{n}\right)}^{2}}{n-1}}$. Thus, it follows that equality occurs in this case if and only if *D* is a symmetric $\Delta^{+}$-regular digraph with three distinct signless Laplacian eigenvalues, which are $q(D)=2\Delta^{+},\frac{a}{n}+\theta$ and $\frac{a}{n}-\theta$.

Conversely, it is easy to see that equality occurs in (3.1) for the digraphs mentioned in the statement of the theorem. This completes the proof. □

We note that the problem of characterizing the connected graphs with three distinct signless Laplacian eigenvalues is well studied and some papers can be found in the literature in this direction. For recent developments we refer to [14] and the references therein.

Proceeding similarly as in Theorem 3.1 and making use of the lower bound given in Theorem 2.6, we obtain the following upper bound for the signless Laplacian energy of a digraph *D*.

Theorem 3.2 *Let D be a digraph of order n with a arcs. Let*$\Delta^{+}$*be the maximum out-degree,*$Zg^{+}(D)$*be the first out-degree Zagreb index and*$c_{2}^{i}$*be the number of closed walks of length* 2 *at vertex* $v_{i}$ *of D. Then*

(3.5) $$E_{SL}(D)\leq 2\Delta^{+}-\frac{a}{n}+\sqrt{\left(n-1)(Zg^{+}(D)+a(1-\frac{a}{n})-{\left(\beta -\frac{a}{n}\right)}^{2}\right)}.$$

*For a strongly connected digraph D, equality occurs in* (3.5) *if and only if* $D={\overset{↔}{K}}_{n}$ *or* $D=\overset{↔}{G}$ *is* $\Delta^{+}$*-regular digraph with three distinct signless Laplacian eigenvalues, given by* $q(D)=2\Delta^{+},\frac{a}{n}+\theta$ *and* $\frac{a}{n}-\theta$*, where* $\theta =\sqrt{\frac{Zg^{+}(D)+a(1-\frac{a}{n})-{\left(\beta -\frac{a}{n}\right)}^{2}}{n-1}}$ *and* $\beta =\sqrt{\frac{\sum_{i=1}^{n}{\left(d_{i}^{+}+c_{2}^{\left(i\right)}\right)}^{2}}{n}}$*.*

The following Arithmetic-Geometric mean inequality can be found in [17].

Lemma 3.3 *If*$a_{1},a_{2},\ldots ,a_{n}$*are non-negative numbers, then*

$$n\left[\frac{1}{n}\sum_{j=1}^{n}a_{j}-{\left(\prod_{j=1}^{n}a_{j}\right)}^{\frac{1}{n}}\right]\leq n\sum_{j=1}^{n}a_{j}-{\left(\sum_{j=1}^{n}\sqrt{a_{j}}\right)}^{2}\leq n(n-1)\left[\frac{1}{n}\sum_{j=1}^{n}a_{j}-{\left(\prod_{j=1}^{n}a_{j}\right)}^{\frac{1}{n}}\right].$$

*Moreover equality occurs if and only if*$a_{1}=a_{2}=\cdots =a_{n}$*.*

The following result gives bounds for the signless Laplacian energy of a digraph *D*, in terms of order *n*, the number of arcs, the first out-degree Zagreb index and the determinant of the matrix $Q(D)-\frac{a}{n}I_{n}$.

Theorem 3.4 *Let D be a digraph of order*n≥3*with a arcs having first out-degree Zagreb index*$Zg^{+}(G)$*and maximum out-degree*$\Delta^{+}$*. Then*

$$E_{SL}(D)\leq 2\Delta^{+}-\frac{a}{n}+\sqrt{(n-2)(\gamma_{1}-{\left(\frac{c_{2}}{n}\right)}^{2})+(n-1){\left(\frac{n}{c_{2}}\right)}^{\frac{2}{n-1}}\gamma_{2}}$$

*and*

$$E_{SL}(D)\geq \frac{c_{2}}{n}+\sqrt{|\gamma_{1}-a+c_{2}|-{\left(2\Delta^{+}-\frac{a}{n}\right)}^{2}+(n-1)(n-2){\left(2\Delta^{+}-\frac{a}{n}\right)}^{\frac{-2}{n-1}}\gamma_{2}},$$

*where*$\gamma_{1}=Zg^{+}(G)+a(1-\frac{a}{n})$*and*$\gamma_{2}=|\det(Q(D)-\frac{a}{n}I_{n}){|}^{\frac{2}{n-1}}$*. Equality occurs in both the inequalities if and only if*$D≅{\overset{↔}{K}}_{n}$*or*$D=\overset{↔}{G}$*is a*$\Delta^{+}$*-regular digraph with three distinct signless Laplacian eigenvalues,*$q(D)=\Delta^{+}$*and the other two eigenvalues with absolute value*$\sqrt{\frac{Zg^{+}(D)+a(1-\frac{a}{n})-{\left(\frac{c_{2}}{n}\right)}^{2}}{n-1}}$*.*

Proof Replacing *n* by n−1 and setting $a_{j}=|\alpha_{j}{|}^{2}$, for j=2,…,n in Lemma 3.3, we have

$$\alpha \leq (n-1)\sum_{j=2}^{n}|\alpha_{j}{|}^{2}-{\left(\sum_{j=2}^{n}|\alpha_{j}|\right)}^{2}\leq (n-2)\alpha ,$$

that is,

(3.6) $$\alpha \leq (n-1)\sum_{j=2}^{n}|\alpha_{j}{|}^{2}-{\left(E_{SL}(G)-|\alpha_{1}|\right)}^{2}\leq (n-2)\alpha ,$$

where

$$\alpha =(n-1)\left[\frac{1}{n-1}\sum_{j=2}^{n}|\alpha_{j}{|}^{2}-{\left(\prod_{j=2}^{n}|\alpha_{j}{|}^{2}\right)}^{\frac{1}{n-1}}\right]=\sum_{j=2}^{n}|\alpha_{j}{|}^{2}-(n-1){\left(\prod_{j=2}^{n}|\alpha_{j}|\right)}^{\frac{2}{n-1}}=\sum_{j=2}^{n}|\alpha_{j}{|}^{2}-\frac{\left(n-1\right)}{|\alpha_{1}{|}^{\frac{2}{n-1}}}|\det(Q(D)-\frac{a}{n}I_{n}){|}^{\frac{2}{n-1}}.$$

Using inequality (3.2) and the value of *α*, it follows from the left inequality of (3.6) that

$${\left(E_{SL}(D)-|\alpha_{1}|\right)}^{2}\leq (n-2)\sum_{j=2}^{n}|\alpha_{j}{|}^{2}+\frac{\left(n-1\right)}{|\alpha_{1}{|}^{\frac{2}{n-1}}}|\det(Q(D)-\frac{a}{n}I_{n}){|}^{\frac{2}{n-1}},$$

that is,

(3.7) $$E_{SL}(D)\leq \alpha_{1}+\sqrt{(n-2)(\gamma_{1}-\alpha_{1}^{2})+(n-1)\alpha_{1}^{\frac{-2}{n-1}}|\det(Q(G)-\frac{a}{n}I_{n}){|}^{\frac{2}{n-1}}},$$

where $\gamma_{1}=Zg^{+}(D)+a(1-\frac{a}{n})$ and $\alpha_{1}\geq 0$. Since, by inequality (3.4), $q(D)\leq 2\Delta^{+}$, it follows that $|\alpha_{1}|=\alpha_{1}=q(D)-\frac{a}{n}\leq 2\Delta^{+}-\frac{a}{n}$. Also, by Theorem 2.4, we have $q(D)\geq \frac{a+c_{2}}{n}$, giving that $|\alpha_{1}|=q(D)-\frac{a}{n}\geq \frac{c_{2}}{n}$. Using the inequalities $\alpha_{1}\geq \frac{c_{2}}{n}$ and $\alpha_{1}\leq 2\Delta^{+}-\frac{a}{n}$ in (3.7) we get the first inequality.

Again using the value of *α*, it follows from the right inequality of (3.6) that

(3.8) $${\left(E_{SL}(D)-|\alpha_{1}|\right)}^{2}\geq \sum_{j=2}^{n}|\alpha_{j}{|}^{2}+(n-1)(n-2)|\alpha_{1}{|}^{\frac{-2}{n-1}}|\det(Q(D)-\frac{a}{n}I_{n}){|}^{\frac{2}{n-1}}.$$

Note that in [23] it is shown that $\sum_{j=1}^{n}\alpha_{j}^{2}=\sum_{j=1}^{n}{\left(d_{i}^{+}-\frac{a}{n}\right)}^{2}+c_{2}=Zg^{+}(D)-\frac{a^{2}}{n}+c_{2}=\gamma_{1}-a+c_{2}$, giving that $\sum_{j=2}^{n}|\alpha_{j}{|}^{2}\geq |\sum_{j=2}^{n}\alpha_{j}^{2}|=|\gamma_{1}-a+c_{2}|$. This together with inequality (3.8) gives that

(3.9) $$E_{SL}(G)\geq \alpha_{1}+\sqrt{|\gamma_{1}-a+c_{2}|-\alpha_{1}^{2}+(n-1)(n-2)\alpha_{1}^{\frac{-2}{n-1}}|\det(Q(D)-\frac{a}{n}I_{n}){|}^{\frac{2}{n-1}}}.$$

Now, using the inequalities $\alpha_{1}\geq \frac{c_{2}}{n}$ and $\alpha_{1}\leq 2\Delta^{+}-\frac{a}{n}$ in (3.9) we get the second inequality.

Equality occurs in the first inequality if and only if

(i) $T=(t_{ij})$ is a diagonal matrix,
(ii) the equality in Lemma 3.3 holds true,
(iii) the equality in $q(D)\leq 2\Delta^{+}$ holds true and
(iv) the equality in $q(D)\geq \frac{a+c_{2}}{n}$ holds true.

From Schur's unitary triangularization theorem [16], we know that $T=(t_{ij})$ is a diagonal matrix if and only if Q(D) is a normal matrix. By (3.4), equality for a strongly connected digraph *D* occurs in $q(D)\leq 2\Delta^{+}$, if and only if *D* is a $\Delta^{+}$-out-degree regular digraph. By Theorem 2.4 equality for a strongly connected digraph *D* occurs in $q(D)\geq \frac{a+c_{2}}{n}$, if and only if $D=\overset{↔}{G}$, where each connected component of *G* is a *r*-regular graph with $r=\frac{a+c_{2}}{n}$. Combining these observations it follows from Lemma 3.3 that equality occurs in the first inequality if and only if $D=\overset{↔}{G}$, where each connected component of *D* is a $\Delta^{+}$-regular digraph and $\left|\alpha_{2}|=|\alpha_{3}|=\cdots =|\alpha_{n}\right|$. Now, proceeding similar to Theorem 3.1, the result follows in this case.

On the other hand equality occurs in the second inequality if and only if equality occurs in $\sum_{j=2}^{n}|\alpha_{j}{|}^{2}\geq |\sum_{j=2}^{n}\alpha_{j}^{2}|$ and equality occurs in (ii), (iii) and (iv). Equality occurs in $\sum_{j=2}^{n}|\alpha_{j}{|}^{2}\geq |\sum_{j=2}^{n}\alpha_{j}^{2}|$, if and only if $\alpha_{2}^{2}=\alpha_{3}^{2}=\cdots =\alpha_{n}^{2}$. From this and above discussion the result now follows.

Conversely, it is easy to see that equality occurs in each of the inequalities for the mentioned cases. This completes the proof. □

If we apply the lower bound given by Theorem 2.6, we obtain the following result for the signless Laplacian energy of a digraph *D*.

Theorem 3.5 *Let D be a digraph of order*n≥3*having a arcs and having first out-degree Zagreb index*$Zg^{+}(G)$*and maximum out-degree*$\Delta^{+}$*. We have*

$$E_{SL}(D)\leq 2\Delta^{+}-\frac{a}{n}+\sqrt{(n-2)(\gamma_{1}-{\left(\beta -\frac{a}{n}\right)}^{2})+(n-1){\left(\beta -\frac{a}{n}\right)}^{\frac{-2}{n-1}}\gamma_{2}}$$

*and*

$$E_{SL}(D)\geq \beta -\frac{a}{n}\;\;+\sqrt{|\gamma_{1}-a+c_{2}|-{\left(2\Delta^{+}-\frac{a}{n}\right)}^{2}+(n-1)(n-2){\left(2\Delta^{+}-\frac{a}{n}\right)}^{\frac{-2}{n-1}}\gamma_{2}},$$

*where*$\gamma_{1}=Zg^{+}(G)+a(1-\frac{a}{n})$*and*$\gamma_{2}=|\det(Q(D)-\frac{a}{n}I_{n}){|}^{\frac{2}{n-1}}$*. The equalities in both inequalities hold true if and only if*$D≅{\overset{↔}{K}}_{n}$*or*$D=\overset{↔}{G}$*is a*$\Delta^{+}$*-regular digraph with three distinct signless Laplacian eigenvalues,*$q(D)=\Delta^{+}$*and the other two eigenvalues with absolute value*$\sqrt{\frac{Zg^{+}(D)+a(1-\frac{a}{n})-{\left(\beta -\frac{a}{n}\right)}^{2}}{n-1}}$*.*

## 4 Concluding remarks

If we take $D=\overset{↔}{G}$, in which $\overset{↔}{G}$ is the symmetric digraph corresponding to the underlying graph *G* of the digraph *D*, the results obtained in Sections 2 and 3 become the corresponding results for the signless Laplacian spectral radius λ(Q(G)) and the signless Laplacian energy QE(G) of the graph *G*. Our results are a generalization of the known results for the signless Laplacian spectral radius and the signless Laplacian energy of a graph *G*.

## Declarations

### Author contribution statement

Hilal A. Ganie, Yilun Shang: Conceived and designed the experiments; Performed the experiments; Analyzed and interpreted the data; Contributed reagents, materials, analysis tools or data; Wrote the paper.

### Funding statement

This research did not receive any specific grant from funding agencies in the public, commercial, or not-for-profit sectors.

### Data availability statement

No data was used for the research described in the article.

### Declaration of interests statement

The authors declare no conflict of interest.

### Additional information

No additional information is available for this paper.
